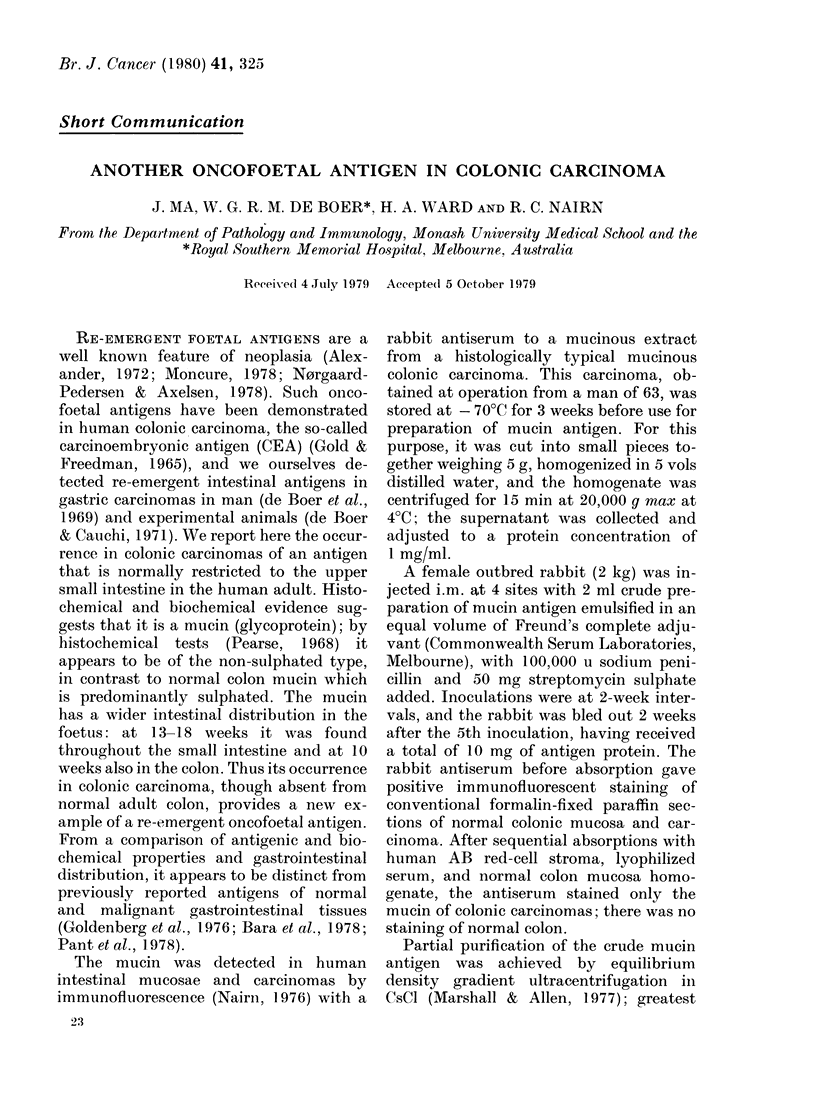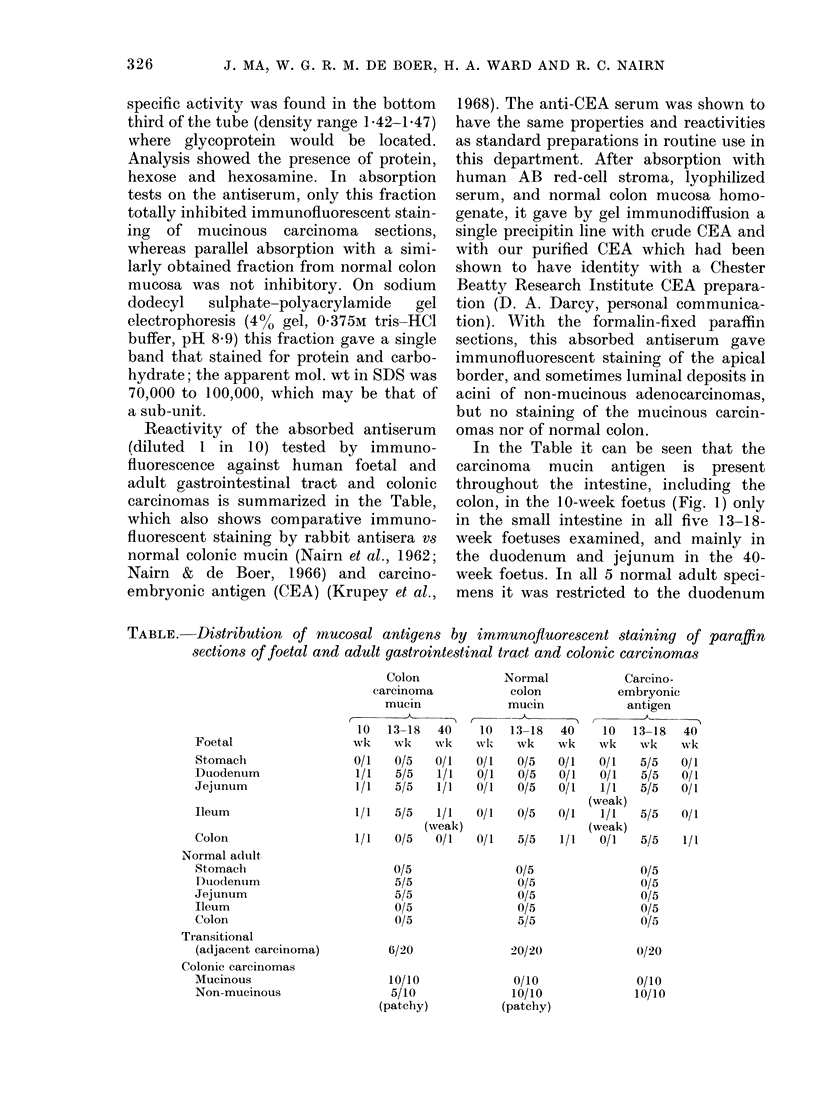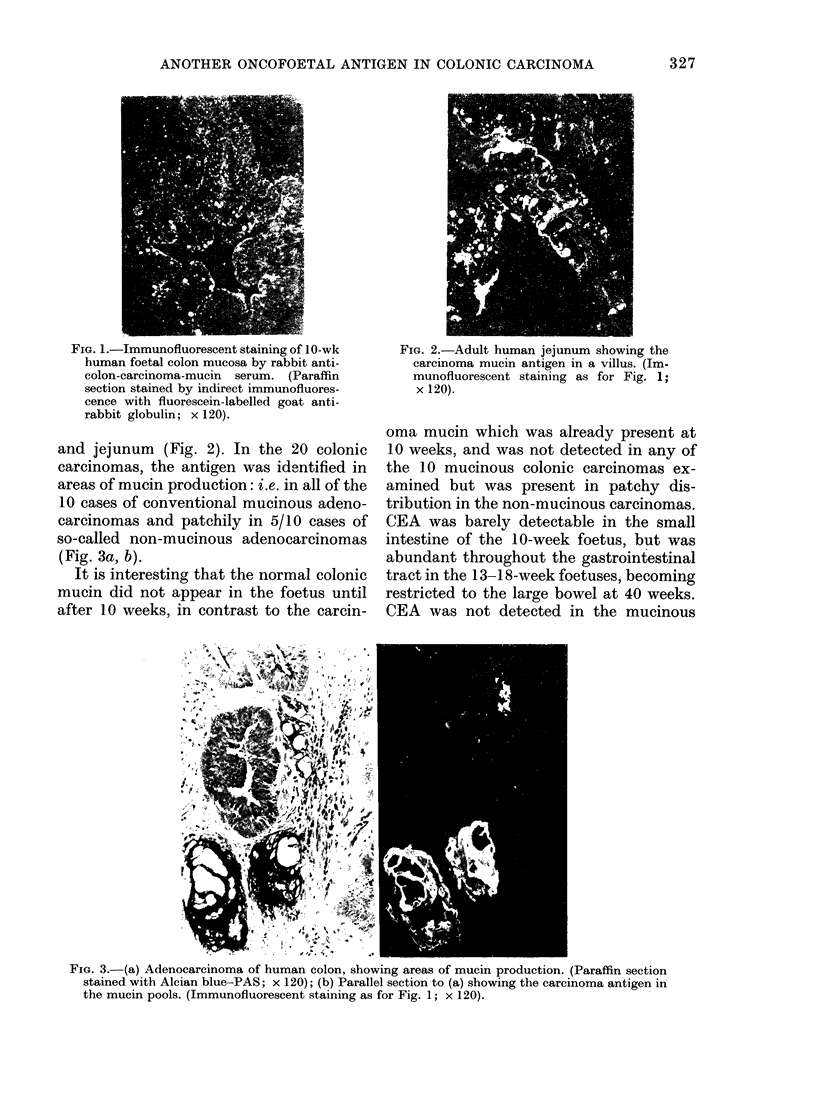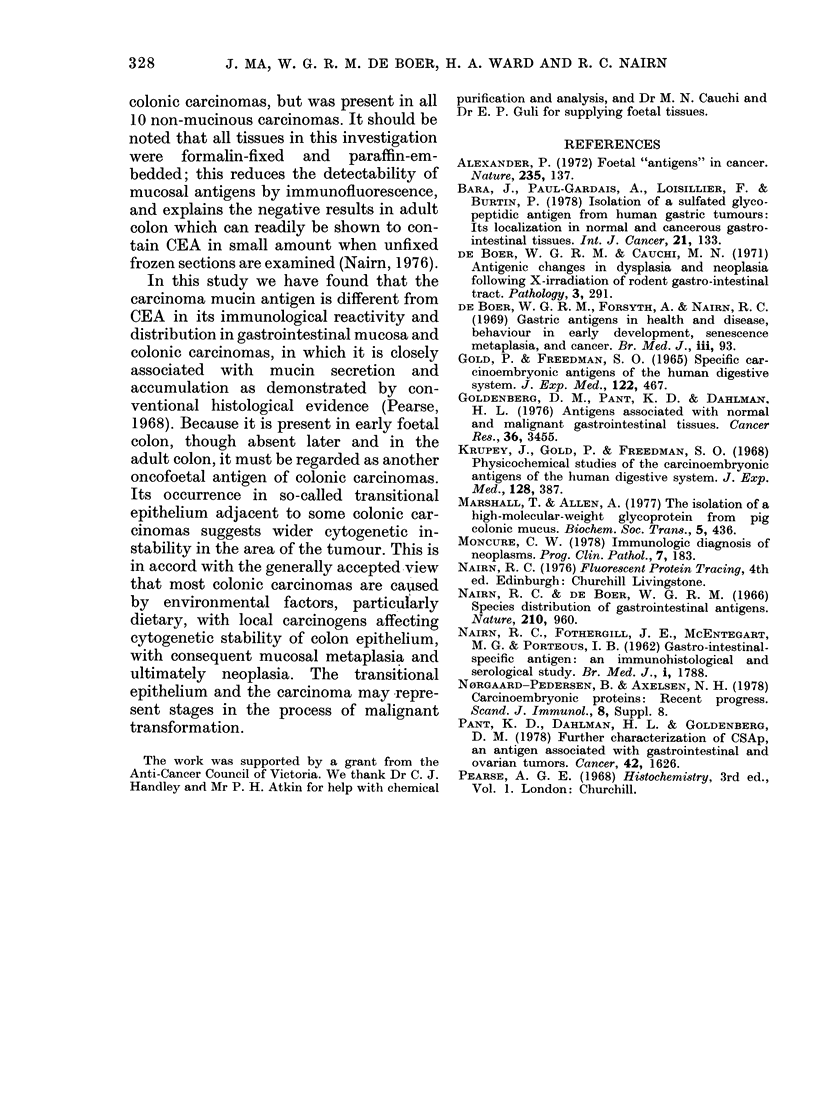# Another oncofoetal antigen in colonic carcinoma.

**DOI:** 10.1038/bjc.1980.49

**Published:** 1980-02

**Authors:** J. Ma, W. G. de Boer, H. A. Ward, R. C. Nairn

## Abstract

**Images:**


*Br. J. Cancer* (1980) **41**, 325

***Short Communication***

### ANOTHER ONCOFOETAL ANTIGEN IN COLONIC CARCINOMA

J. MA, W. G. R. M. DE BOER*, H. A. WARD AND R. C. NAIRN

*From the Department of Pathology and Immunology, Monash University Medical School and the *Royal Southern Memorial Hospital, Melbourne, Australia*

Received 4 July 1979 Accepted 5 October 1979

RE-EMERGENT FOETAL ANTIGENS are a well known feature of neoplasia (Alexander, 1972; Moncure, 1978; Nørgaard-Pedersen & Axelsen, 1978). Such oncofoetal antigens have been demonstrated in human colonic carcinoma, the so-called carcinoembryonic antigen (CEA) (Gold & Freedman, 1965), and we ourselves detected re-emergent intestinal antigens in gastric carcinomas in man (de Boer *et al.*, 1969) and experimental animals (de Boer & Cauchi, 1971). We report here the occurrence in colonic carcinomas of an antigen that is normally restricted to the upper small intestine in the human adult. Histochemical and biochemical evidence suggests that it is a mucin (glycoprotein); by histochemical tests (Pearse, 1968) it appears to be of the non-sulphated type, in contrast to normal colon mucin which is predominantly sulphated. The mucin has a wider intestinal distribution in the foetus: at 13–18 weeks it was found throughout the small intestine and at 10 weeks also in the colon. Thus its occurrence in colonic carcinoma, though absent from normal adult colon, provides a new example of a re-emergent oncofoetal antigen. From a comparison of antigenic and biochemical properties and gastrointestinal distribution, it appears to be distinct from previously reported antigens of normal and malignant gastrointestinal tissues (Goldenberg *et al.*, 1976; Bara *et al.*, 1978; Pant *et al.*, 1978).

The mucin was detected in human intestinal mucosae and carcinomas by immunofluorescence (Nairn, 1976) with a rabbit antiserum to a mucinous extract from a histologically typical mucinous colonic carcinoma. This carcinoma, obtained at operation from a man of 63, was stored at $-70°C$ for 3 weeks before use for preparation of mucin antigen. For this purpose, it was cut into small pieces together weighing 5 g, homogenized in 5 vols distilled water, and the homogenate was centrifuged for 15 min at 20,000 $g$ *max* at 4°C; the supernatant was collected and adjusted to a protein concentration of 1 mg/ml.

A female outbred rabbit (2 kg) was injected i.m. at 4 sites with 2 ml crude preparation of mucin antigen emulsified in an equal volume of Freund's complete adjuvant (Commonwealth Serum Laboratories, Melbourne), with 100,000 u sodium penicillin and 50 mg streptomycin sulphate added. Inoculations were at 2-week intervals, and the rabbit was bled out 2 weeks after the 5th inoculation, having received a total of 10 mg of antigen protein. The rabbit antiserum before absorption gave positive immunofluorescent staining of conventional formalin-fixed paraffin sections of normal colonic mucosa and carcinoma. After sequential absorptions with human AB red-cell stroma, lyophilized serum, and normal colon mucosa homogenate, the antiserum stained only the mucin of colonic carcinomas; there was no staining of normal colon.

Partial purification of the crude mucin antigen was achieved by equilibrium density gradient ultracentrifugation in CsCl (Marshall & Allen, 1977); greatest specific activity was found in the bottom third of the tube (density range 1·42–1·47) where glycoprotein would be located. Analysis showed the presence of protein, hexose and hexosamine. In absorption tests on the antiserum, only this fraction totally inhibited immunofluorescent staining of mucinous carcinoma sections, whereas parallel absorption with a similarly obtained fraction from normal colon mucosa was not inhibitory. On sodium dodecyl sulphate–polyacrylamide gel electrophoresis (4% gel, 0·375M tris–HCl buffer, pH 8·9) this fraction gave a single band that stained for protein and carbohydrate; the apparent mol. wt in SDS was 70,000 to 100,000, which may be that of a sub-unit.

Reactivity of the absorbed antiserum (diluted 1 in 10) tested by immunofluorescence against human foetal and adult gastrointestinal tract and colonic carcinomas is summarized in the Table, which also shows comparative immunofluorescent staining by rabbit antisera *vs* normal colonic mucin (Nairn *et al.*, 1962; Nairn & de Boer, 1966) and carcinoembryonic antigen (CEA) (Krupey *et al.*, 1968). The anti-CEA serum was shown to have the same properties and reactivities as standard preparations in routine use in this department. After absorption with human AB red-cell stroma, lyophilized serum, and normal colon mucosa homogenate, it gave by gel immunodiffusion a single precipitin line with crude CEA and with our purified CEA which had been shown to have identity with a Chester Beatty Research Institute CEA preparation (D. A. Darcy, personal communication). With the formalin-fixed paraffin sections, this absorbed antiserum gave immunofluorescent staining of the apical border, and sometimes luminal deposits in acini of non-mucinous adenocarcinomas, but no staining of the mucinous carcinomas nor of normal colon.

In the Table it can be seen that the carcinoma mucin antigen is present throughout the intestine, including the colon, in the 10-week foetus (Fig. 1) only in the small intestine in all five 13–18-week foetuses examined, and mainly in the duodenum and jejunum in the 40-week foetus. In all 5 normal adult specimens it was restricted to the duodenum

TABLE.—*Distribution of mucosal antigens by immunofluorescent staining of paraffin sections of foetal and adult gastrointestinal tract and colonic carcinomas*

| | Colon carcinoma mucin | | | Normal colon mucin | | | Carcino-embryonic antigen | | |
|---|---|---|---|---|---|---|---|---|---|
| Foetal | 10 wk | 13–18 wk | 40 wk | 10 wk | 13–18 wk | 40 wk | 10 wk | 13–18 wk | 40 wk |
| Stomach | 0/1 | 0/5 | 0/1 | 0/1 | 0/5 | 0/1 | 0/1 | 5/5 | 0/1 |
| Duodenum | 1/1 | 5/5 | 1/1 | 0/1 | 0/5 | 0/1 | 0/1 | 5/5 | 0/1 |
| Jejunum | 1/1 | 5/5 | 1/1 | 0/1 | 0/5 | 0/1 | 1/1 (weak) | 5/5 | 0/1 |
| Ileum | 1/1 | 5/5 | 1/1 (weak) | 0/1 | 0/5 | 0/1 | 1/1 (weak) | 5/5 | 0/1 |
| Colon | 1/1 | 0/5 | 0/1 | 0/1 | 5/5 | 1/1 | 0/1 | 5/5 | 1/1 |
| Normal adult | | | | | | | | | |
| Stomach | | 0/5 | | | 0/5 | | | 0/5 | |
| Duodenum | | 5/5 | | | 0/5 | | | 0/5 | |
| Jejunum | | 5/5 | | | 0/5 | | | 0/5 | |
| Ileum | | 0/5 | | | 0/5 | | | 0/5 | |
| Colon | | 0/5 | | | 5/5 | | | 0/5 | |
| Transitional (adjacent carcinoma) | | 6/20 | | | 20/20 | | | 0/20 | |
| Colonic carcinomas | | | | | | | | | |
| Mucinous | | 10/10 | | | 0/10 | | | 0/10 | |
| Non-mucinous | | 5/10 (patchy) | | | 10/10 (patchy) | | | 10/10 | |

FIG. 1.—Immunofluorescent staining of 10-wk human foetal colon mucosa by rabbit anti-colon-carcinoma-mucin serum. (Paraffin section stained by indirect immunofluorescence with fluorescein-labelled goat anti-rabbit globulin; ×120).

FIG. 2.—Adult human jejunum showing the carcinoma mucin antigen in a villus. (Immunofluorescent staining as for Fig. 1; ×120).

and jejunum (Fig. 2). In the 20 colonic carcinomas, the antigen was identified in areas of mucin production: *i.e.* in all of the 10 cases of conventional mucinous adenocarcinomas and patchily in 5/10 cases of so-called non-mucinous adenocarcinomas (Fig. 3*a*, *b*).

It is interesting that the normal colonic mucin did not appear in the foetus until after 10 weeks, in contrast to the carcinoma mucin which was already present at 10 weeks, and was not detected in any of the 10 mucinous colonic carcinomas examined but was present in patchy distribution in the non-mucinous carcinomas. CEA was barely detectable in the small intestine of the 10-week foetus, but was abundant throughout the gastrointestinal tract in the 13–18-week foetuses, becoming restricted to the large bowel at 40 weeks. CEA was not detected in the mucinous

FIG. 3.—(a) Adenocarcinoma of human colon, showing areas of mucin production. (Paraffin section stained with Alcian blue–PAS; ×120); (b) Parallel section to (a) showing the carcinoma antigen in the mucin pools. (Immunofluorescent staining as for Fig. 1; ×120).

colonic carcinomas, but was present in all 10 non-mucinous carcinomas. It should be noted that all tissues in this investigation were formalin-fixed and paraffin-embedded; this reduces the detectability of mucosal antigens by immunofluorescence, and explains the negative results in adult colon which can readily be shown to contain CEA in small amount when unfixed frozen sections are examined (Nairn, 1976).

In this study we have found that the carcinoma mucin antigen is different from CEA in its immunological reactivity and distribution in gastrointestinal mucosa and colonic carcinomas, in which it is closely associated with mucin secretion and accumulation as demonstrated by conventional histological evidence (Pearse, 1968). Because it is present in early foetal colon, though absent later and in the adult colon, it must be regarded as another oncofoetal antigen of colonic carcinomas. Its occurrence in so-called transitional epithelium adjacent to some colonic carcinomas suggests wider cytogenetic instability in the area of the tumour. This is in accord with the generally accepted view that most colonic carcinomas are caused by environmental factors, particularly dietary, with local carcinogens affecting cytogenetic stability of colon epithelium, with consequent mucosal metaplasia and ultimately neoplasia. The transitional epithelium and the carcinoma may represent stages in the process of malignant transformation.

The work was supported by a grant from the Anti-Cancer Council of Victoria. We thank Dr C. J. Handley and Mr P. H. Atkin for help with chemical purification and analysis, and Dr M. N. Cauchi and Dr E. P. Guli for supplying foetal tissues.

### REFERENCES

ALEXANDER, P. (1972) Foetal "antigens" in cancer. *Nature*, **235**, 137.

BARA, J., PAUL-GARDAIS, A., LOISILLIER, F. & BURTIN, P. (1978) Isolation of a sulfated glycopeptidic antigen from human gastric tumours: Its localization in normal and cancerous gastrointestinal tissues. *Int. J. Cancer*, **21**, 133.

DE BOER, W. G. R. M. & CAUCHI, M. N. (1971) Antigenic changes in dysplasia and neoplasia following X-irradiation of rodent gastro-intestinal tract. *Pathology*, **3**, 291.

DE BOER, W. G. R. M., FORSYTH, A. & NAIRN, R. C. (1969) Gastric antigens in health and disease, behaviour in early development, senescence metaplasia, and cancer. *Br. Med. J.*, **iii**, 93.

GOLD, P. & FREEDMAN, S. O. (1965) Specific carcinoembryonic antigens of the human digestive system. *J. Exp. Med.*, **122**, 467.

GOLDENBERG, D. M., PANT, K. D. & DAHLMAN, H. L. (1976) Antigens associated with normal and malignant gastrointestinal tissues. *Cancer Res.*, **36**, 3455.

KRUPEY, J., GOLD, P. & FREEDMAN, S. O. (1968) Physicochemical studies of the carcinoembryonic antigens of the human digestive system. *J. Exp. Med.*, **128**, 387.

MARSHALL, T. & ALLEN, A. (1977) The isolation of a high-molecular-weight glycoprotein from pig colonic mucus. *Biochem. Soc. Trans.*, **5**, 436.

MONCURE, C. W. (1978) Immunologic diagnosis of neoplasms. *Prog. Clin. Pathol.*, **7**, 183.

NAIRN, R. C. (1976) *Fluorescent Protein Tracing*, 4th ed. Edinburgh: Churchill Livingstone.

NAIRN, R. C. & DE BOER, W. G. R. M. (1966) Species distribution of gastrointestinal antigens. *Nature*, **210**, 960.

NAIRN, R. C., FOTHERGILL, J. E., MCENTEGART, M. G. & PORTEOUS, I. B. (1962) Gastro-intestinal-specific antigen: an immunohistological and serological study. *Br. Med. J.*, **i**, 1788.

NØRGAARD-PEDERSEN, B. & AXELSEN, N. H. (1978) Carcinoembryonic proteins: Recent progress. *Scand. J. Immunol.*, **8**, Suppl. 8.

PANT, K. D., DAHLMAN, H. L. & GOLDENBERG, D. M. (1978) Further characterization of CSAp, an antigen associated with gastrointestinal and ovarian tumors. *Cancer*, **42**, 1626.

PEARSE, A. G. E. (1968) *Histochemistry*, 3rd ed., Vol. 1. London: Churchill.